# Epigenetic Alterations in Alzheimer’s Disease

**DOI:** 10.3389/fnbeh.2015.00347

**Published:** 2015-12-17

**Authors:** Jose V. Sanchez-Mut, Johannes Gräff

**Affiliations:** Neuroepigenetics Laboratory – UPGRAEFF, Brain Mind Institute, School of Life Sciences, École Polytechnique Fédérale de LausanneLausanne, Switzerland

**Keywords:** Alzheimer’s disease, epigenetics, neuroepigenetics, DNA methylation, histone acetylation, histone methylation, histone phosphorylation

## Abstract

Alzheimer’s disease (AD) is the major cause of dementia in Western societies. It progresses asymptomatically during decades before being belatedly diagnosed when therapeutic strategies have become unviable. Although several genetic alterations have been associated with AD, the vast majority of AD cases do not show strong genetic underpinnings and are thus considered a consequence of non-genetic factors. Epigenetic mechanisms allow for the integration of long-lasting non-genetic inputs on specific genetic backgrounds, and recently, a growing number of epigenetic alterations in AD have been described. For instance, an accumulation of dysregulated epigenetic mechanisms in aging, the predominant risk factor of AD, might facilitate the onset of the disease. Likewise, mutations in several enzymes of the epigenetic machinery have been associated with neurodegenerative processes that are altered in AD such as impaired learning and memory formation. Genome-wide and locus-specific epigenetic alterations have also been reported, and several epigenetically dysregulated genes validated by independent groups. From these studies, a picture emerges of AD as being associated with DNA hypermethylation and histone deacetylation, suggesting a general repressed chromatin state and epigenetically reduced plasticity in AD. Here we review these recent findings and discuss several technical and methodological considerations that are imperative for their correct interpretation. We also pay particular focus on potential implementations and theoretical frameworks that we expect will help to better direct future studies aimed to unravel the epigenetic participation in AD.

## Introduction

The term “epigenetics” was introduced by the developmental biologist Conrad Hal Waddington (1905–1975) in the early 1940s. He defined epigenetics as the branch of biology that studies the causal interactions between genes and their products, which bring the phenotype into being (Waddington, 1942, 2012). This general idea was later narrowed and defined as the science that studies the heritable traits resulting from changes in a chromosome without altering the DNA sequence (Berger et al., 2009). Although nowadays widely accepted, this definition is – strictly speaking – a conception of developmental and cancer researchers, who perceive epigenetics as a way to transmit phenotypic characteristics to daughter cells. Yet, such conception represents a major problem for other disciplines like neurosciences, since neurons do not divide and, accordingly, nothing happening in neurons would be considered epigenetics. As “epigenetic newcomers,” neuroscientists have only recently started to add their viewpoints toward this perception, but due to the emerging importance of epigenetics in the nervous system, this has already stimulated a profound revision of the perception of epigenetics. Currently, epigenetics is rather considered as the study of structural changes of the chromatin that modify the phenotype without altering the genotype (Jaenisch and Bird, 2003), independently of whether the cells divide or not.

Yet, where does this recent interest of neuroscientists in epigenetics or, in other words, in neuroepigenetics originate from? Probably one of the main reasons is because epigenetic mechanisms provide a platform for integrating different *inputs* and for generating adaptive long-lasting *outputs*. This capacity of epigenetics touches upon the very fundament of neuroscience, providing a potential substrate for memory allocation, and for articulating the hypothesis of gene × environment interaction associated with many multifactorial diseases such as Parkinson’s and Alzheimer’s disease (AD), amyotrophic lateral and multiple sclerosis, and even epilepsy (Urdinguio et al., 2009). As a matter of fact, it is known that epigenetic mechanisms participate in the processes of learning and memory formation (Levenson and Sweatt, 2005; Gräff and Tsai, 2013a; Zovkic et al., 2013; Guzman-Karlsson et al., 2014; Jarome et al., 2014; Woldemichael et al., 2014), and that – on the other end of the spectrum – life style (Fraga et al., 2005), aging (Heyn et al., 2012), nutrition (Cooney et al., 2002), and environmental toxins (Anway et al., 2005) associated with AD can modify the epigenetic makeup and might thereby contribute to the pathophysiology of AD (Cacabelos and Torrellas, 2014; Coppede, 2014; Bennett et al., 2015).

## Epigenetic Mechanisms

At the molecular level, it is generally accepted that epigenetics encompasses two main mechanisms: the direct methylation of the DNA, and the modification of the proteins that package the DNA, the histones. Chromatin remodelers and non-coding RNAs can also participate in the regulation of the chromatin but, because they are not considered purely epigenetic mechanisms, are not included in this review (for a further discussion about these topics see Magistri et al., 2012; Langst and Manelyte, 2015). Here, we first provide a description of the functioning of these two epigenetic modifications in general, before moving to their implication in neuroscience, and in particular, in AD.

### DNA Methylation

DNA methylation has thus far been the most studied epigenetic modification. It mainly consists of the addition of a methyl group at cytosines that precede guanines (so-called CpG dinucleotides). These dinucleotides are underrepresented in the genome and tend to accumulate in CpG-dense regions (so-called CpG islands, or CGI) although around 95% of CpGs are scattered through all the genome without showing any type of aggregation. As a general view, CpGs in non-CGI and CGIs tend to be fully methylated and non-methylated, respectively, with a negligible amount of CpGs being partially methylated (Vinson and Chatterjee, 2012).

Historically, DNA methylation has been considered an epigenetic mark of repression, since seminal experiments have shown that the genomic insertion of exogenous DNA results in active transcription only with non-methylated DNA (Pollack et al., 1980; Wigler et al., 1981; Stein et al., 1982), and since CGI hypermethylation has been recurrently associated with the silencing of tissue-specific genes and X inactivation (Straussman et al., 2009). In fact, the existence of CGI together with the bimodal pattern of DNA methylation has evoked the idea that genes can be switch ON an OFF by controlling the DNA methylation of their CGIs. This idea has been predominant in the last years. However, it is now apparent that the reality is much more complex since around 70% of annotated genes contain CGI regions in their promoters (Deaton and Bird, 2011) and most of them are non-methylated. Furthermore, CGI and non-CGI promoter-containing genes show specific particularities, with the former being constitutively expressed and displaying low evolution rates and relaxed use of transcription start sites (TSS), while the latter is characterized by a more restricted pattern of expression, higher evolution rate, and strongly defined TSS usage (Tang and Epstein, 2007). Thus, DNA methylation cannot be the main driver of gene expression in these regions.

Importantly, DNA is not randomly distributed in the nucleus but associated to histones forming the nucleosomes (Schones et al., 2008). The distribution and the compaction of these nucleosomes determines the chromatin structure and thereby the access of the transcriptional machinery to the DNA (Schones et al., 2008). Accordingly, CGIs favors the access of the DNA polymerase and gene expression by constituting a rigid structure that complicates the wrapping of DNA and nucleosome positioning (Ramirez-Carrozzi et al., 2009). Therefore, CGI are not mere platforms for controlling gene expression by DNA methylation, but such effect depends on the nearby sequence and thus, three-dimensional chromatin context. In general, DNA methylation in gene promoters is associated with lower levels of expression (Kelly et al., 2012) whereas in gene bodies, it favors gene expression (Guenther et al., 2007).

The enzymes that carry out the active DNA methylation, the so called DNA methyltransferases (DNMTs), are all associated with the nucleosomes (Jeong et al., 2009), which reinforces the idea that DNA methylation and nucleosome positioning are intimately related. Three different DNMTs have been identified: DNMT1 – necessary for the maintenance of DNA methylation patterns during cell division – and DNMT3A and DNMT3B, both involved in establishing *de novo* patterns of DNA methylation during development and cell fate determination. Interestingly, DNMTs also show high levels of expression in post-mitotic neurons (Guo et al., 2014a), suggesting that their importance in the adult brain is beyond the classical developmental point of view. A deficit of these enzymes can cause passive DNA demethylation (Rhee et al., 2002), but DNA can also be actively demethylated by the action of several enzymatic reactions. These include the 10–11 translocation proteins (TET), which mediate the oxidation of 5-methylcytosines (5mC) to 5-hydroxymethylcytosine (5hmC), and later on to 5-formilcytosine (5fC) and 5-carboxycytosine (5caC); and the thymine-DNA glycosylases (TDG), which causes the final excision and conversion to cytosines (Kohli and Zhang, 2013).

### Newly Identified DNA Methylation Marks

The recently developed techniques of deep-sequencing have documented an unexpected high prevalence of 5hmC and 5fC in brain (Lister et al., 2013; Varley et al., 2013; Guo et al., 2014a,b, Kozlenkov et al., 2014). In spite of that, it is still under discussion whether 5hmC and 5fC constitute new epigenetic marks *per se* or if they are just intermediate states of the DNA demethylation (Hahn et al., 2014). In the brain, around ∼80% of cytosines in CpG sites are methylated (5mC), whereas ∼8% are hydroxyl-methylated (5hmC), ∼0.8% are formyl-methylated (5fC), and even less are carboxyl-methylated (5caC). These data reflect a high prevalence of the intermediate states, in special for 5hmC, which has been used as an argument to emphasize the specific role of 5hmC in epigenetic signaling (Globisch et al., 2010; Song et al., 2011; Lister et al., 2013; Wen et al., 2014), which together with 5fC/5caC is enriched in enhancers and gene bodies of highly transcribed genes (Song et al., 2011, 2013; Shen et al., 2013; Wen et al., 2014; Raiber et al., 2015).

Also, a certain degree of DNA methylation outside of CpG dinucleotides has recently been reported. The so-called non-CpG DNA methylation mainly occurs in the context of CpA dinucleotides (Lister et al., 2009; Yan et al., 2011; Ziller et al., 2011) and is particularly prevalent in the brain where it accounts for ∼25% of all cytosine modifications (Lister et al., 2013; Guo et al., 2014a). Similarly to 5mC and 5hmC, non-CpG methylation also tends to occur in gene bodies of highly transcribed genes (Lister et al., 2013; Guo et al., 2014a).

### Histone Modifications

As aforementioned, nucleosomes are important components of the chromatin structure and their positioning is influenced by DNA methylation and sequence context. Notwithstanding, nucleosomes are primarily regulated by posttranslational modifications that tend to occur in the N-terminal tail of histone proteins (Bowman and Poirier, 2015). The most studied of these are histone acetylation and methylation, which occur as a consequence of the antagonistic activity of histone acetyltransferases (HATs) and deacetylates (HDACs), and of histone methyltransferases (HMTs) and demethylases (HDMTs), respectively, as well as histone phosphorylation, which is mediated by the opposing action of protein kinases and phosphatases. Further, more recently discovered posttranslational modifications include ADP-ribosylation, ubiquitylation, sumoylation, crotonylation, propionylation, deiminiation and *O*-GlcNAcylation, which are also the consequence of a similar set of enzyme complexes.

These modifications can take place on different amino acids. For instance, lysines can be acetylated; mono-, di-, or trimethylated; and mono- or polyubiquitylated; arginines can be deaminated; mono-, symmetrically or asymmetrically dimethylated, and mono- or poly-ADP-ribosylated; serines and threonines can be phosphorylated and *O*-GlcNAcylated; glutamates can be mono- or poly-ADP-ribosylated; prolines can be isomerized; and tyrosines can be phosphorylated (Hanes, 2014; Xu et al., 2014; Bowman and Poirier, 2015).

### Histone Acetylation

Depending on their identity, posttranslational histone modifications can have different effects on gene expression. In general, histone acetylation is associated with increased gene activity (Kouzarides, 2007), in part because it diminishes the basic charge of histones and thereby reduces the electrostatic interaction with the negatively charged DNA chains. As a result, nucleosome compaction is relaxed facilitating the access of the transcriptional machinery to the DNA (Li and Reinberg, 2011). In line, histone acetylation is enriched in promoter and gene bodies of active genes (Wang et al., 2008b). Some of the most studied lysine acetylation modifications include the acetylation of lysine 9 on histone 3 (H3K9ac) and H3K27ac in gene promoters and H3K4ac and H4K12ac in gene bodies, among others (Wang et al., 2008b).

Three families of HATs – GNAT, MYST, and CBP/p300 – are responsible of the acetylation of these amino acids, and the specificity of these modifications depend on their association with other regulatory proteins (Bannister and Kouzarides, 2011). In line, although many acetylation sites has been individually described, they tend to occur in combinations (Baker, 2011). Zinc-dependent class I, II and IV HDACs as well as the NAD-dependent class III HDACs, the sirtuins, antagonize the activity of HATs. Similarly to the HATs, HDAC proteins show a low level of specificity that is mainly regulated by the interaction with other non-catalytic proteins and complexes (Yang and Seto, 2007).

### Histone Methylation

As opposed to acetylation, the effect of histone methylation depends on both the type of modification and the residue on which it occurs. As an example, the mono-methylation of lysine 27 of histone 3 (H3K27me1) is enriched in promoters of active genes, whereas the tri-methylation of the same amino acid (H3K27me3) is mainly found in repressed genes (Wang et al., 2008b). Also in contrast to histone acetylation, the enzymes that regulate histone methylation show a high degree of specificity. Two classes of lysine HMTs are at play: SET domain and non-SET domain-containing HMTs, which install histone methylation at particular lysines to different degrees. For instance, three different members of the SET HMTs perform H4K20 methylation in its mono-, di-, or trimethylated form (Liu et al., 2010; Qi et al., 2010). The activity of the HMTs is antagonized by two classes of HDMTs – LSD1- and Jumonji-related HDMTs – that also show a high degree of specificity. For instance, LSD1 demethylates the monomethylation and dimethylation of H3K4 and H3K9, but not their trimethylation (Bannister and Kouzarides, 2011).

### The Histone Code

The sum of different histone modifications, their intricate system of regulation, and the almost infinite number of possible combinations draws a complex landscape of histone modifications that – considering that chromatin is composed by hundreds of millions of nucleosomes – can epigenetically encode an immense amount of information. This idea has inspired the hypothesis of the “histone code,” which suggests that by using different combinations of histone modifications cells can regulate specific and distinct functional chromatin outputs (Strahl and Allis, 2000; Jenuwein and Allis, 2001). This intriguing view has attracted a lot of attention in last years that has undoubtedly helped to push the development of the field. In spite of that, recent chromatin immunoprecipitation (ChIP) sequencing experiments have suggested that the complexity behind such histone code seems smaller than previously estimated. In fact, a high degree of redundancy between different histone marks has been reported (Wang et al., 2008b) and algorithms have recognized recurrent combinations of histone modifications that can be grouped into discrete chromatin states, which account for most of gene transcription variance (Baker, 2011). Obviously, this might be a simplification of the reality, and the numbers of identified chromatin states strongly depends of the level of technical resolution applied, but importantly, it underlines that from all possible combinations only few of them ever really occur together, suggesting that the histone code is simpler than anticipated, which would facilitate the task of epigeneticists considerably.

### Epigenetic Crosstalk

Importantly, DNA methylation and histone modifications are not isolated phenomena, and proteins that recognize and regulate both epigenetic marks are orchestrated in multiprotein complexes. For instance, MeCP2 and KAISO proteins, which recognizes methylated DNA, are also associated with HDAC1 and HDAC3, linking DNA methylation and histone deacetylation (Suzuki et al., 2003; Yoon et al., 2003); likewise, other DNA methylation readers such as UHRF1 are known to interact with the H3K9 HMT G9A, associating, in this case, DNA methylation with H3K9 methylation (Meilinger et al., 2009; Rottach et al., 2010). Thus, the concept of a “histone code” should be expanded to one of an epigenetic code, which also applies to the nervous system (Gräff and Mansuy, 2008). Besides, the different histone marks also interact with each other, as it attested by, for instance, the fact that the HAT GCN5 is also able to recognize H3K4me3 (Guillemette et al., 2011), which can help to understand the apparent redundancy of some epigenetic marks (for a more comprehensive view of different epigenetic marks crosstalk see Du and Patel, 2014).

Beyond such crosstalk, the different epigenetic marks are ensembles interpreted by so-called chromatin remodelers – SWI/SNF, NuRD and ISWI families (for a detailed description see Langst and Manelyte, 2015) – which modify the presence, composition, and nucleosome positioning regulating the chromatin accessibility. Therefore, epigenetic modifications should be understood as chromatin states instead of isolated modifications independently associated with particular functions, which seem to have a special relevance in the nervous system.

## Neuroepigenetics

The nervous system is a highly specialized system in which millions of cells are organized in different structures with characteristic epigenetic and expression profiles that are associated with particular functions (Xin et al., 2010; Ko et al., 2013; Sanchez-Mut et al., 2013). It is in the nervous system where three out of four genes are expressed (Johnson et al., 2009), where most splicing variants are transcribed (Stamm et al., 2000; Xu et al., 2002; Yeo et al., 2004) and most miRNAs are synthesized (Cao et al., 2006). Also, it is in the nervous system, where the expression patterns of cells show the highest degree of heterogeneity, with more than 70% of genes being expressed in less than 20% of the cells of the entire brain (Lein et al., 2007). Owing to this complexity, the transcription machinery faces a formidable challenge in the nervous system, and, as a consequence, is also highly sensitive to epigenetic perturbations.

Accordingly, the importance of epigenetics in the functioning of the nervous system is underlined by the fact that mutations in epigenetic genes cause severe mental disorders (Berdasco and Esteller, 2013). For example, mutations in genes that establish epigenetic marks, such as *DNMT1*, *NSD1*, *NSD2*, or *CBP* cause hereditary sensory autonomic neuropathy with dementia (HSAN1), Sotos, Wolf–Hirschhorn and Rubinstein–Taybi syndromes, respectively. Similarly, mutations in genes that remove epigenetic marks, such as KDM5C, recognize them, such as *MeCP2*, or are in charge of their integration, such as SWI/SNF proteins, are associated with X-linked mental retardation, Rett syndrome and Coffin-Siris syndromes respectively (Urdinguio et al., 2009; Gasser and Li, 2011; Sanchez-Mut et al., 2012; Berdasco and Esteller, 2013).

But one the most important findings that supports the importance of epigenetics in the functioning of the brain has been the discovery that neuronal activity *per se* modifies DNA methylation and histone modifications patterns, and further, that learning and memory depend on these epigenetic changes (Levenson et al., 2004; Miller and Sweatt, 2007; Guan et al., 2009; Ma et al., 2009; Gupta et al., 2010; Miller et al., 2010; Guo et al., 2011; Gräff et al., 2012; Zovkic et al., 2013). For instance, neuronal activity induces the expression of DNMT3A2, TET1, and TET3 (Guo et al., 2011; Oliveira et al., 2012; Rudenko et al., 2013; Li et al., 2014b) and the shuttling of HDAC4 to the nucleus (Sando et al., 2012), whereas the depletion of DNMTs 1 and 3a, of the HATs KAT2A and KAT2B, of the HDMT neuronal specific isoform LSD1, and of the HMTs GLP and G9A as well as the increased expression of MeCP2, of HDACs 2, 3, 4, and 5 impair learning and memory formation (Guan et al., 2009; Feng et al., 2010; Kramer et al., 2011; McQuown et al., 2011; Na et al., 2012; Sando et al., 2012; Agis-Balboa et al., 2013; Kerimoglu et al., 2013; Morris et al., 2013; Stilling et al., 2014; Wang et al., 2015). Furthermore, several HDAC inhibitors, such as valproic acid, sodium butyrate and others, potentiate learning and memory formation in different paradigms and animal models (Gräff and Tsai, 2013a), as well as in different neurological diseases such as Alzheimer, Parkinson’s, and Huntington diseases (Zhang et al., 2013). Therefore, it is evident that neuronal activity as well as learning and memory engage and to some degree depend on numerous epigenetic players, and that epigenetic perturbations not only impair brain normal functioning but also are associated with many neurological diseases, including AD.

## Epigenetic Alterations in AD

Alzheimer’s disease is the main cause of dementia in Western societies, where it affects 17% of people older than 65 years and 50% older than 85 years (Alzheimer’s Association, 2010). AD is a neurodegenerative disorder characterized by a progressive decline in mental abilities, neuronal loss and accumulation of two types of protein aggregates, amyloid plaques and neurofibrillary tangles (NFT; Cummings, 2004). Amyloid plaques are mainly constituted by aggregates of the amyloid-β (Aβ)-peptide, itself being a consequence of the cleavage of the amyloid precursor protein (APP) by β- and γ-secretases, while NFT are aggregates of hyperphosphorylated TAU protein. These two hallmarks are unequivocally associated with AD, but whether they are cause or consequence, and the mechanisms leading to their formation and propagation are poorly understood.

**FIGURE 1:**
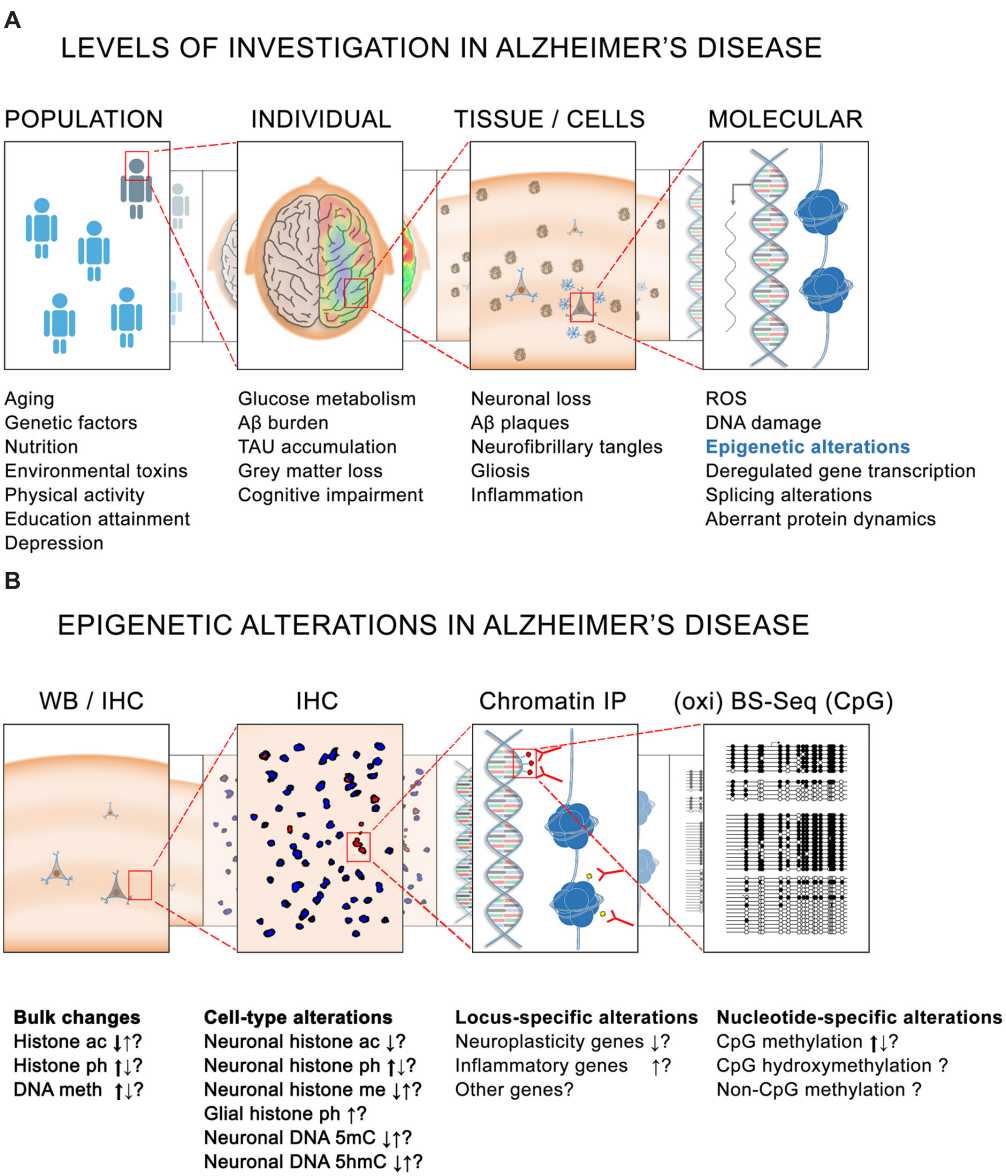
**Epigenetics in Alzheimer’s disease (AD).** **(A)** Different levels of investigation in AD pathology. At the population level, several genetic and non-genetic factors contribute to the risk for developing the disease. At the level of the individual, several pathophysiological characteristics such as altered glucose metabolism are observed in the brain of cognitively impaired AD patients. Associated with these alterations are – at the tissue and cellular level – yet other pathological hallmarks such as the presence of amyloid plaques and neurofibrillary tangles. Finally, at the intracellular level, higher levels of reactive oxygen species (ROS) and DNA damage, together with dysregulated gene transcription, splicing alterations and aberrant protein dynamics are also believed to be implicated in the onset and development of the pathology. **(B)** Similarly, epigenetic alterations have been reported in AD at different levels. Bulk histone acetylation (ac), phosphorylation (ph), and methylation (me) changes as well as DNA methylation (5mC) and hydroxymethylation (5hmC) alterations have been reported in AD tissues by IHC and WB. Major tendencies for these changes (as observed by several studies) are indicated by thick arrows. Locus-specific alterations mainly causing a repression of neuroplasticity genes and an activation of inflammatory genes have also been observed by ChIP using antibodies against histone modifications. Greater resolution is also possible for DNA methylation analysis in which nucleotide-specific alterations can be detected by oxi- and BS-sequencing. BS, bisulfite sequencing; ChIP, chromatin immunoprecipitation; CpG, cytosine-guanine dinucleotide; IHC, immunohistochemistry; IP, immunoprecipitation; WB, western blotting.

It is known that genetic and non-genetic factors contribute to the development of AD. Rare mutations in three genes – *APP*, *PSEN1*, and *PSEN2* – are associated with 1% of AD (Chouraki and Seshadri, 2014) and other frequent genetic variants such as *APOE-E4* can account for up to 20% of total cases of the disease (Mayeux and Stern, 2012). In total, the heritability for AD is estimated to explain between one half and two thirds of total AD cases (Ertekin-Taner, 2007), the other third/half being attributable to non-genetic risk factors in which epigenetics mechanisms are supposedly involved, namely diabetes mellitus, hypertension, obesity, physical inactivity, depression, smoking and low educational attainment (**Figure 1A**) (Kivipelto and Mangialasche, 2014).

By far the predominant risk factor for AD is aging itself, since AD only appears in late adulthood, and the risk to develop the disease doubles every 5 years after age 65 (Kawas et al., 2000). Importantly, epigenetic mechanisms have also been suggested to be a major force of aging (Chouliaras et al., 2012, 2013b; Heyn et al., 2012) and similar epigenetic alterations have also been described in AD (**Figure 1B**) (Cacabelos and Torrellas, 2014; Coppede, 2014; Bennett et al., 2015). But prior to outlining the evidence of an epigenetic implication in AD, it is important to mention that this question has been addressed from different (technical) perspectives, and that the obtained results strongly depend of the experimental approaches, the samples analyzed, and the techniques used. Some of these studies are based on cell lines, others on animal models, and yet others on human post-mortem tissue, sometimes with a limited sample number at their disposal. Equally diverse are the techniques being used for determining the levels of DNA methylation, which range from the use of DNA methylation sensitive restriction enzymes, to antibodies that specifically recognize DNA methylation modifications, and to a direct reading of DNA methylation by bisulfite-sequencing (**Figure 1B**).

### DNA Methylation

#### Global DNA Methylation Changes

In general, the use of cell lines, independently of the technique used, suggest that AD is associated with lower levels of DNA methylation. For instance, the glioblastoma cell line H4 harboring the Swedish mutation of *APP* (K670M/N671L double mutation segregating in a Swedish family), which causes an increase in Aβ production (Citron et al., 1992; Mullan et al., 1992; Haass et al., 1995), shows a general tendency toward hypomethylation as measured by DNA microarrays following bisulfite conversion (Sung et al., 2011). Similarly, treatment of the neuronal-like cell line SH-SY5Y with conditioned media obtained from cells harboring the Indiana mutation (V717F mutation identified by a group of Indiana) – associated with higher Aβ levels (Murrell et al., 1991; Suzuki et al., 1994) –induced a general DNA hypomethylation as measured by DNA methylation-sensitive antibodies (Hodgson et al., 2013). In line, brain microvascular endothelial cells subjected to high levels of synthetic Aβ show lower levels of DNA methylation as measured by high-performance liquid chromatography (HPLC) (Chen et al., 2009). Nevertheless, conversely to the previously discussed observations, IMR-32 neuroblastoma cells subjected to high levels of synthetic Aβ do not show significant alterations in DNA methylation as measured by DNA microarrays (Taher et al., 2014).

In mouse models of AD, global levels of DNA methylation have been less studied and, to our knowledge, only one work has addressed this question. Cong et al. (2014) used cortical samples of APPswe/PS1dE9 mice – harboring the Swedish *APP* mutation in combination with the deletion of the exon 9 of the *PSEN1* resulting in increases Aβ formation (Borchelt et al., 1997) – and immunoprecipitation of the DNA using DNA methylation specific antibodies (Methylated DNA immunoprecipitation: MeDIP) followed by the hybridization of the resulting DNA to promoter microarrays. Following this approach, around 10% of analyzed genes (2346 of 20404 promoter genes contained in the array) showed higher levels of DNA methylation in the APPswe/PS1dE9 mice than in the controls, and no hypomethylated genes were reported (Cong et al., 2014). Therefore, conversely to what occurs in cell line models of the disease, at least one AD mouse model displays higher levels of DNA methylation.

The study of human post-mortem samples has not helped to solve this apparent discrepancy. Using antibodies that recognize methylated DNA, a loss of DNA methylation has been observed in the entorhinal cortex (Mastroeni et al., 2010) and the hippocampus of post-mortem samples of AD (Chouliaras et al., 2013a). Conversely, other studies using the same technique have reported no differences in the entorhinal cortex (Lashley et al., 2015) or even gains of DNA methylation in the frontal cortex (Coppieters et al., 2014), the temporal cortex (Coppieters et al., 2014) and the hippocampus of AD samples (Bradley-Whitman and Lovell, 2013). In much the same manner, ELISA 5mC assays of the entorhinal cortex of AD patients (Lashley et al., 2015) as well as DNA methylation microarrays in frontal cortex (Bakulski et al., 2012) have not shown significant DNA methylation differences.

A similarly confusing scenario is also emerging from the study of the DNA hydroxymethylation in AD. Higher levels of DNA hydroxymethylation have been reported in 3xTg-AD mice – harboring the *APP* Swedish, *PSEN1* M146L, and P301L *TAU* mutations and resulting in Aβ formation and TAU phosphorylation (Oddo et al., 2003) – using specific 5hmC antibodies (Cadena-del-Castillo et al., 2014), but lower levels have been observed using the same technique in the human frontal, entorhinal, and temporal cortex (Condliffe et al., 2014; Coppieters et al., 2014) as well as in the hippocampus of post-mortem AD samples (Chouliaras et al., 2013a), with no significant differences observed in entorhinal cortex using 5hmC specific ELISA assays (Lashley et al., 2015).

It is important to mention that, in all these studies, the magnitude of DNA methylation and hydroxymethylation changes, and the number of samples analyzed, are relatively small, and as a consequence the results can easily be influenced by differences in the analyzed regions (Ladd-Acosta et al., 2007; Xin et al., 2010; Hernandez et al., 2011; Lee et al., 2011; Davies et al., 2012; Sanchez-Mut et al., 2013), interindividual variability (Turan et al., 2010; Heyn et al., 2013) and experimental fluctuations. As a result, if DNA methylation and hydroxymethylation differences are present in AD, these are likely to be either small or to be associated with only discrete regions of the genome. The study of disease-discordant twins has been crucial for unraveling the epigenetic component of common diseases (Bell and Spector, 2011), but unfortunately, only a single couple of monozygotic twins discordant for AD has been studied so far. There, AD was associated with a loss of DNA methylation and an increase of DNA hydroxymethylation (Mastroeni et al., 2009; Chouliaras et al., 2013a).

#### Gene-Specific DNA Methylation Changes

Attempts to determine whether specific genetic regions or particular genes are altered in AD have initially focused on genes previously associated with the disease – *APP*, *PSEN1*, and *TAU* genes –, and similarly to the global tendencies, no conclusive evidences have emerged from these studies.

In spite of some reports suggesting a hypomethylation in the promoter of *APP* in the temporal cortex of AD (West et al., 1995) and aging (Tohgi et al., 1999), studies using higher sample numbers have not been able to find differences in frontal cortex, parietal cortex, and hippocampus of AD patients (Yoshikai et al., 1990; Wang et al., 2008a; Barrachina and Ferrer, 2009). Similarly, studies aimed to determine whether *PSEN1* might also be epigenetically deregulated in AD have not been conclusive. Methyl groups are directly assimilated from diet, which itself is frequently deficient in aging and AD (Ford and Almeida, 2012; Hinterberger and Fischer, 2013). Indeed, in the case of dietary depletion, the *PSEN1* promoter can become hypomethylated in TgCRND8 – harboring the Swedish and V717F Indiana *APP* mutations (Chishti et al., 2001) – and APPswe/PS1dE9 AD models (Fuso et al., 2011; Li et al., 2015). Similar findings have been obtained in SK-N-BE neuroblastoma cell line using vitamin B6 and B12 deficient media (Fuso et al., 2005). However, although mechanistically possible, *PSEN1* hypomethylation has not been observed in frontal cortex and hippocampus of AD samples (Wang et al., 2008a; Barrachina and Ferrer, 2009). And finally, no significant differences in DNA methylation in the frontal cortex or the hippocampus of post-mortem AD samples have been observed in the promoter of *TAU* (Barrachina and Ferrer, 2009). Therefore, it seems that at least these three classical AD-associated genes are not epigenetically dysregulated in AD at the DNA methylation level, which might indicate that DNA methylation changes do not play a role in AD, or that genetic and non-genetic forms of AD might be the results of alterations in a different subset of genes. As a consequence, unbiased genome wide screening are also starting to be performed.

#### Genome-Wide DNA Methylation Changes

Unfortunately, genome-wide control-case comparisons have not been more conclusive, with almost every single study reporting a different subset of altered genes which might reflect that current approaches are still immature (**Table 1**). However, the combination of genome-wide strategies with longitudinal studies of AD patients and mouse models yields more consistent data. Two different genes have been reported to be hypermethylated by two independent groups, namely *Sorbin And SH3 Domain Containing 3* (*SORBS3*) (Siegmund et al., 2007; Sanchez-Mut et al., 2014) and *Ankyrin 1* (*ANK1*) (De Jager et al., 2014; Lunnon et al., 2014). These results were obtained in an age-dependent DNA methylation study and a genome-wide DNA methylation screening in two different AD mouse models – APPswe/dE9 and 3xTg-AD – and later validated in the frontal cortex of human post-mortem AD samples, as well as from two genome-wide DNA methylation screenings in several human brain regions in differentially AD-affected samples respectively. Equally relevant seems to be the hypermethylation of the gene *Insulin-Like Growth Factor Binding Protein 7* (*IGFBP7*), which is sustained by consistent changes in DNA methylation in the APPPS1-21 AD mouse model – harboring the Swedish *APP* mutation in combination with the L166P *PSEN1* mutation (Radde et al., 2006) – and in human frontal cortex samples (Agbemenyah et al., 2014). Lastly, the hypermethylation of *Dual Specificity Phosphatase 22* (*DUSP22*), similarly to *ANK1*, correlates with the severity of the disease and was demonstrated modify TAU phosphorylation and cell viability *in vitro* (Sanchez-Mut et al., 2014).

**Table 1:** Differentially DNA methylated genes reported in Alzheimer’s disease (AD).

Gene	Change	Genomic region	Technique	RNA	Organism	Brain region	References
ANK1	Increase	Gene body	BS-array and pyrosequencing	NA	Human	Entorhinal, temporal, and prefrontal cortex	Lunnon et al., 2014
	Increase	Gene body	BS-array	Decrease	Human	Entorhinal, temporal, and prefrontal cortex	De Jager et al., 2014
BIN1	Increase	Downstream (Intergenic)	BS-array	NA	Human	Entorhinal, temporal, and prefrontal cortex	De Jager et al., 2014
BDNF	Decrease	Promoter	MSRE-PCR	Decrease	Human	Frontal cortex	Rao et al., 2012
CDH3	Increase	Gene body	BS-array	Increase	Human	Entorhinal, temporal, and prefrontal cortex	De Jager et al., 2014
COX2	Decrease	Promoter	MSRE-PCR	NA	Human	Frontal cortex	Rao et al., 2012
CREB	Increase	Promoter	MSRE-PCR	NA	Human	Frontal cortex	Rao et al., 2012
DUSP22	Increase	Promoter	BS-array and pyrosequencing	Decrease	Human	Hippocampus	Sanchez-Mut et al., 2014
FOXK1	Increase	Gene body	BS-array	NA	Human	Entorhinal, temporal, and prefrontal cortex	De Jager et al., 2014
F2RL2	Increase	Promoter	BS-array and pyrosequencing	Decrease	APP/PS1 and 3Xtg-AD	Frontal cortex	Sanchez-Mut et al., 2013
HMHA1 (ABCA7)	Increase	Gene body/Promoter (Downstream)	BS-array	NA	Human	Entorhinal, temporal, and prefrontal cortex	De Jager et al., 2014
HOXA3	Increase	Gene body	BS-array	NA	Human	Entorhinal, temporal, and prefrontal cortex	De Jager et al., 2014
IGFBP7	Increase	Promoter	MeDIP	Increase in APPPS1-21	APPPS1-21 and human	Frontal cortex	Agbemenyah et al., 2014
ITPRIPL2	Increase	Gene body	BS-array	NA	Human	Entorhinal, temporal, and prefrontal cortex	De Jager et al., 2014
KDM2B	Increase	Gene body	BS-array	NA	Human	Entorhinal, temporal, and prefrontal cortex	De Jager et al., 2014
NFKB	Decrease	Promoter	MSRE-PCR	Increase	Human	Frontal cortex	Rao et al., 2012
PCNT (DIP2)	Increase	Gene body (Upstream)	BS-array	NA (increase)	Human	Entorhinal, temporal, and prefrontal cortex	De Jager et al., 2014
RHBDF2	Increase	Gene body	BS-array	Increase	Human	Entorhinal, temporal, and prefrontal cortex	De Jager et al., 2014
SLC2A1	Increase	Upstream (Intergenic)	BS-array	NA	Human	Entorhinal, temporal, and prefrontal cortex	De Jager et al., 2014
SORBS3	Increase	Promoter	MethyLight PCR	NA	Human	Entorhinal, temporal, and prefrontal cortex	Siegmund et al., 2007
	Increase	Promoter	BS-array and pyrosequencing	Decrease	APP/PS1, 3Xtg-AD and Human	Frontal cortex	Sanchez-Mut et al., 2013
SPG7 (RPL13)	Increase	Gene body/Promoter (Upstream)	BS-array	NA (decrease)	Human	Entorhinal, temporal, and prefrontal cortex	De Jager et al., 2014
SPTBN4	Increase	Promoter	BS-array and pyrosequencing	Decrease	APP/PS1, 3Xtg-AD and Human	Frontal cortex	Sanchez-Mut et al., 2013
SYP	Increase	Promoter	MSRE-PCR	NA	Human	Frontal cortex	Rao et al., 2012
S100A2	Decrease	Promoter	MethyLight PCR	NA	Human	Frontal cortex	Siegmund et al., 2007
TBXA2R	Increase	Promoter	BS-array and pyrosequencing	Decrease	APP/PS1, 3Xtg-AD and Human	Frontal cortex	Sanchez-Mut et al., 2013
TMEM59	Decrease	Promoter	BS-array	Increase	APPPS1-21 and Human	Frontal cortex	Bakulski et al., 2012
WDR81 (SERPINF1 and SERPINF2)	Increase	Gene body (Upstream)	BS-array	NA (decrease and increase)	Human	Entorhinal, temporal, and prefrontal cortex	De Jager et al., 2014


*BS-array, bisulfite-modified DNA based arrays, MSRE-PCR, methylation-sensitive restriction enzyme-PCR, MeDIP, Methylated DNA immunoprecipitation. NA, not available.*

Nonetheless, it has to be noted that these correlations do not necessarily reflect a causal relation with the disease, and might even be the consequence of secondary alterations. This is particularly important for the ones observed in mouse models, since these models have already a genetic predisposition to develop AD pathology. Also, another limitation of these studies is that they did not distinguish between different cell populations, the proportions of which are already altered in AD (AD being a neurodegenerative disease associated with a prominent gliosis and a specific loss of neurons (Serrano-Pozo et al., 2011), and which present distinct epigenetic profiles (Iwamoto et al., 2011; Labonte et al., 2013; Lister et al., 2013). Therefore, although promising, these results should be considered with caution since they will require further validations using cell-type specific studies.

### Histone Modifications

#### Global Histone Acetylation Changes

Contrary to DNA methylation, histone modifications have been less studied in AD and evidences linking histone modification alterations with AD are mainly indirect. The few studies that, have found that several HDAC inhibitors exert a protective effect in AD, improving dendritic spine density, and facilitating learning and memory formation in different mouse models of the disease (Fischer et al., 2007; Francis et al., 2009; Ricobaraza et al., 2009, 2012; Zhang and Schluesener, 2013; Rumbaugh et al., 2015), although the precise mechanisms by which the HDAC inhibitors work remain to be determined. Furthermore, HDAC2 was found to be elevated with age in mice and humans (Chouliaras et al., 2013b; Singh and Thakur, 2014), in APP/PS1 (Gonzalez-Zuniga et al., 2014), p25/Cdk5 – harboring the Cdk5 activator *p25* transgene that induces TAU phosphorylation and neurodegeneration (Cruz et al., 2006) – and 5xFAD AD mouse models – harboring the Swedish, I716V Florida, and V717I London *APP* mutations in combination with the M146L and L286L *PSEN1* mutations with induce Aβ formation and neurodegeneration (Oakley et al., 2006) – as well as in the hippocampus and entorhinal cortex of post-mortem human AD samples (Gräff et al., 2012). In line, it has been shown that HDAC2 is able to differentially bind and regulate the expression of several learning and neuroplasticity-related genes, but that its viral-mediated depletion or its specific pharmacological inhibition is sufficient for restoring the synaptic and cognitive deficits observed in p25/Cdk5 mice (Gräff et al., 2012; Wagner et al., 2015). Therefore, there is compelling evidence that HDAC2 is increased in aging and AD, and probably implicated in the associated cognitive decline, although it should be mentioned that a decrease of HDAC2 in AD patients has been also reported by another study (Mastroeni et al., 2011).

Surprisingly, in spite of these evidences, it is still not clear whether basal histone acetylation is altered in AD. Lower (Zhang et al., 2012), equal (Rao et al., 2012; Lu et al., 2014), and higher (Narayan et al., 2015) levels of histone acetylation have been reported for post-mortem AD human samples, whereas no differences have been observed in two different AD mouse models – namely Tg2576 and 3xTg-AD – (Francis et al., 2009; Cadena-del-Castillo et al., 2014), although an increase of H3 and H4 acetylation in primary cultures of the 3xTg-AD mouse has been described by others (Walker et al., 2013). One possible explanation might be that instead of an alteration of the basal levels of histone acetylation, AD might be more related with the incapacity of modifying the epigenetic patterns in certain conditions, such as learning and memory formation, in which HDAC inhibitors that increase histone acetylation would “prime” the levels of histone acetylation and consequently, of gene activity (Gräff and Tsai, 2013a,b; Gräff et al., 2014). In support of this view, the basal levels of H4K12ac in aging remain constant, but when mice are subjected to learning and memory paradigms only young animals are able to increase these levels and not the aged mice (Peleg et al., 2010). Similarly, in Tg2576 AD mice – harboring the Swedish *APP* mutation in combination with the M146V *PSEN1* mutation which results in higher levels of Aβ formation (Chishti et al., 2001) – the global levels of H4 acetylation are not altered, but when mice are subjected to learning and memory paradigms only wild-type animals are able to increase the levels of histone acetylation and not the Tg2576 mice (Francis et al., 2009). Alternatively, although without excluding the previous hypothesis, it could also be possible that histone acetylation alterations occur just in certain loci, which could be more sensitive to HDAC inhibitors, without reflecting general tendencies in the bulk chromatin. To better understand these scenarios, genome wide screenings of histone modifications are starting to be undertaken.

#### Global Tendencies in Other Histone Marks

Less attention has been put on posttranslational modifications of other histone marks, despite some results suggesting that histone phosphorylation might be altered in AD. Namely, the linker histone H1 becomes hyperphosphorylated and accumulates in the cytoplasm of astrocytes and neurons of APPswe/PS1dE9 mice (Duce et al., 2006). Interestingly, H1 is a substrate of p25/Cdk5 that accumulates in AD patients (Patrick et al., 1999; Chang et al., 2012) and is associated with neurodegeneration and cellular damage such as false entrance of cell cycle division (Cruz et al., 2006), and H2A.X phosphorylation (Kim et al., 2008), both pathophysiological characteristics of AD (Ogawa et al., 2003; Myung et al., 2008). In addition to H1 phosphorylation, evidence for H3 phosphorylation has been mixed thus far (Rao et al., 2012; Anderson et al., 2015), suggesting that more studies are necessary for elucidating its role in AD.

The first attempts for studying potential alterations on H3K9 methylation in AD has been equally inconclusive since only three studies have address this question with contradictory results: decreased heterochromatin compaction associated with lower H3K9me2 levels in a TAU Drosophila AD model and human samples (Frost et al., 2014), no significant differences in the heterochromatin of p25/cdk5 Ad mouse model measured by H3K9me3 (Gjoneska et al., 2015), and increased compaction in primary cultures of 3xTg-AD mouse measured by H3K9me2 (Walker et al., 2013).

#### Gene-Specific Histone Alterations

The possibility that in AD specific genes might be posttranslationally modified on their histones has just started to be addressed, and to the extent of our knowledge, only two studies in the p25/Cdk5 AD mouse model addressed this point. In 2012, several neuroplasticity related genes were reported as hypoacetylated and repressed in p25/Cdk5 mice (**Table 2**; Gräff et al., 2012) and, recently, the catalog of deregulated genes and posttranslational modifications has been enormously enlarged (Gjoneska et al., 2015). There, in general, complementary gains and losses of specific marks at discrete loci were observed, explaining the minor global alterations reported in previous studies (Gjoneska et al., 2015). An interesting finding of this study was further a consistent enrichment of active marks (H3K27ac and H3K4me3) in enhancers and promoters of immune and stimulus-response functions coupled with a specific decrease in synapse and learning-associated functions can be observed (Gjoneska et al., 2015).

**Table 2:** Differentially histone acetylated genes in AD.

Gene	Change		Genomic region	Technique	RNA	Organism	Brain region	References
ARC	H3K14, H4K5, H4K12	Decrease	Promoter	ChIP	Decrease	p25/cdk5	Hippocampus	Gräff et al., 2012
BDNF I	H4K5, H4K12	Decrease	Promoter	ChIP	No change	p25/cdk5	Hippocampus	Gräff et al., 2012
BDNF IV	H2BK5, H4K5, H4K12	Decrease	Promoter	ChIP	Decrease	p25/cdk5	Hippocampus	Gräff et al., 2012
	H4 (pan)	Decrease	Promoter	ChIP	Decrease	C57Bl/6J vs APP KO	Prefrontal cortex	Hendrickx et al., 2014
CDK5	H2BK5, H4K5, H4K12	Decrease	Promoter	ChIP	Decrease	p25/cdk5	Hippocampus	Gräff et al., 2012
EGR1	H2BK5, H4K5, H4K12	Decrease	Promoter	ChIP	Decrease	p25/cdk5	Hippocampus	Gräff et al., 2012
	H4K5, H4K12ac	Decrease	Promoter	ChIP	Decrease	C57Bl/6J vs APP KO	Prefrontal cortex	Hendrickx et al., 2014
FOS	H4K5, H4K12ac	Decrease	Promoter	ChIP	Decrease	C57Bl/6J vs APP KO	Prefrontal cortex	Hendrickx et al., 2014
HOMER1	H2BK5, H3K14, H4K5, H4K12	Decrease	Promoter	ChIP	Decrease	p25/cdk5	Hippocampus	Gräff et al., 2012
GLUR1	H3K14, H4K5, H4K12	Decrease	Promoter	ChIP	Decrease	p25/cdk5	Hippocampus	Gräff et al., 2012
GLUR2	H2BK5, H3K14	Decrease	Promoter	ChIP	Decrease	p25/cdk5	Hippocampus	Gräff et al., 2012
NFL	H4K12	Decrease	Promoter	ChIP	Decrease	p25/cdk5	Hippocampus	Gräff et al., 2012
NR2A	H2BK5, H3K14, H4K5, H4K12	Decrease	Promoter	ChIP	Decrease	p25/cdk5	Hippocampus	Gräff et al., 2012
NR2B	H2BK5, H3K14, H4K5, H4K12	Decrease	Promoter	ChIP	Decrease	p25/cdk5	Hippocampus	Gräff et al., 2012
SYP	H2BK5, H3K14, H4K5, H4K12	Decrease	Promoter	ChIP	Decrease	p25/cdk5	Hippocampus	Gräff et al., 2012
SYT1	H4K12	Decrease	Promoter	ChIP	Decrease	p25/cdk5	Hippocampus	Gräff et al., 2012


*ChIP, chromatin immunoprecipitation.*

Similarly to reported DNA methylation alterations in AD, these results probably reflect both changes in cell composition and cell-type-specific changes associated with AD pathology, thereby necessitating cell-specific validations for a better evaluation of their significance in AD.

## Considerations

There is an increasing interest of neuroscientists in epigenetics that is likely to result in a fruitful synergy, which will undoubtedly push the frontiers of both fields: epigenetic researchers have approached neuroscience, and vice versa, neuroscientists have also approached epigenetics. Nonetheless, the following conceptual, methodological, and biological caveats need to be properly addressed from both the epigenetic and the neuroscience point of view in order draw meaningful conclusions from these studies.

### DNA Methylation Marks: Independent or Redundant?

In spite of the abundant literature arguing in favor of the specific roles for the different types of DNA methylation mark, it is still unclear whether they are independent or redundant. 5mC, 5hmC, and 5fC/5caC show similar distribution and association with gene transcription, being mainly enriched in gene bodies and correlated with gene expression (Guenther et al., 2007; Song et al., 2011; Shen et al., 2013; Wu et al., 2014), and when occurring in TSS, associated with gene repression (Guenther et al., 2007; Shen et al., 2013). In line, 5mC and 5hmC are significantly correlated (Lashley et al., 2015). Furthermore, non-CpG methylation tends to occur on gene bodies of highly transcribed genes (Lister et al., 2013; Guo et al., 2014a) and to accumulate in aging (data presented in AD/PD 2015) in the same manner than 5mC and 5hmC (Hernandez et al., 2011; Szulwach et al., 2011). A possible explanation for this might be that CpGs are continuously being methylated and demethylated in promoters of highly transcribed genes questioning their specific effect and explaining the high prevalence and concordance of these DNA methylation marks (Neri et al., 2015). 5mC, 5hmC, and 5fC/5caC are indeed produced in a stepwise manner (Kohli and Zhang, 2013), and it has been suggested that non-CpG methylation could be a consequence of the DNMT3B binding to previously methylated CpG sites (Ramsahoye et al., 2000; Arand et al., 2012, Baubec et al., 2015). Therefore, it is possible that, similarly to the initial high expectations about the histone code, that the complexity behind DNA methylation might be currently overestimated.

Then, it is also worth to mention that different techniques seem to show different scenarios. There is an apparent discrepancy between DNA methylation levels reported by antibody-based immunoprecipitation and by classical bisulfite-dependent modification of DNA, the former usually reporting higher values than the latter (Clark et al., 2012). Furthermore, antibody-based techniques tend to enrich densely modified regions (Pastor et al., 2011) and classical bisulfite DNA modification cannot distinguish between 5mC and 5hmC (Nestor et al., 2010). Therefore, it might be possible that instead of reporting just DNA methylation differences, antibody-based techniques could be reporting a combination of DNA methylation differences and other alterations to the chromatin structure – not necessarily related with the DNA methylation differences – and that the classical bisulfite-dependent modification of DNA might be underestimating the 5mC changes. In line, most of the reported DNA methylation changes based on bisulfite-dependent DNA modifications are gains of methylation, since losses of DNA methylation should be coupled with gains of 5hmC and are consequently masked in this technique. The application of the recently developed oxidative bisulfite-dependent DNA modification could help to resolve these discrepancies since it combines the precision of the classical bisulfite DNA modification with the ability of differentiate between 5mC and 5hmC (Booth et al., 2013).

### HDAC Inhibitors in Learning and AD: Only an Epigenetic Effect?

Similarly, it is also important to consider several constraints when analyzing histone modifications. The beneficial effect of HDAC inhibitors in learning and AD can be interpreted as a proof of the involvement of histone acetylation in these processes (Vogel-Ciernia and Wood, 2012; Gräff and Tsai, 2013a) but, instead, it is just proving the involvement of the inhibited enzyme *per se*, and not necessarily of the acetylation of histones since other non-histones substrates can be also acetylated and deacetylated (Martinez-Redondo and Vaquero, 2013; Li et al., 2014a). For instance, it is known that HDAC6 is elevated in AD patients (Ding et al., 2008), and that its deletion restores the cognitive deficits observed in APPPS1-21 mouse model of AD (Govindarajan et al., 2013), but it is also known that the main effect of this protection is a consequence of the modification of tubulin acetylation (Govindarajan et al., 2013). Similarly, it has been seen that SIRT1 decreases in aging and in AD (Julien et al., 2009; Quintas et al., 2012), and that its restoration protects against neurodegeneration (Kim et al., 2007; Gräff et al., 2013), but again its main effects are associated with non-histone substrates, including PGC-1alpha, p53, and TAU (Kim et al., 2007; Min et al., 2010). Therefore, these studies should be interpreted cautiously not only in light of the particular specificity of the HDAC inhibitor, but also in light of the potential non-histone substrates of the targeted HDAC.

### Epigenetics and Gene Expression, Always Coupled?

Also, it is worth to mention that, in most cases, the interpretation of the effect of epigenetic marks on gene transcription is based on genome-wide comparisons in which significant correlations can be seen, but when single genes are being analyzed a strikingly high level of discrepancy is observed (Lopez-Atalaya and Barco, 2014). In fact, changes in gene transcription can occur independently of epigenetic modifications (Zhang et al., 2014), and changes in epigenetic modifications are not necessarily reflected by changes in gene expression (Lopez-Atalaya and Barco, 2014).

Along these lines, it is important to note that epigenetic mechanisms mainly regulate the chromatin structure, which secondarily modifies the accessibility of the genome to impact on gene regulation. Several epigenetic players also interact with the gene transcription machinery, but it is simplistic to assume that epigenetic changes will completely determine the levels of gene transcription since many other factors are also implied. More likely, epigenetics would be one of the variables of the probabilistic model that finally determines the levels and the magnitude of the potential changes in gene expression. Furthermore, most epigenetic changes occur in enhancer and regulatory regions that are not easily assignable to specific genes (Gjoneska et al., 2015; Sun and Yi, 2015). These assignments are frequently based on distance criteria, which do not necessarily reflect real interactions. In fact, chromatin conformation capture experiments have shown that the majority of regulatory elements do not interact with the nearest genes (Sanyal et al., 2012). Therefore, an important part of long-range epigenetic information is still far from being understood.

### Cause or Consequence?

Finally, it is important to consider that the majority of studies investigating potential epigenetic alterations in AD are based on correlations, which do not necessarily reflect a causal relationship. Therefore, whether epigenetic modifications are driving the chromatin behavior or whether they are just a consequence of other processes happening nearby is still unknown. These studies also require considerable amounts of chromatin, which is achieved, in the best of the cases, by collecting pools of similar cell types. However, unlike in cell lines, this is a particularly difficult feature to achieve in the heterogeneous central nervous system. In fact, every single cell might have its specific epigenetic and expression profile. As a consequence, the pooling of cells could mix different epigenetic patterns, thereby masking potentially important changes and complicating any analysis. Epigenetic AD studies ought thus to be conducted in particular cell types. Recently, the first report of single-cell DNA methylation profiling has just appeared (Guo et al., 2015), raising hope that cell-type specific epigenetic profiling might in the future also become an option for AD.

In spite of these limitations, it is becoming more and more evident that by modifying the chromatin structure, epigenetic mechanisms can shape genome accessibility and thereby have an impact on gene transcription. And that, by doing so, epigenetic changes might provide a molecular substrate for “chromatin memories” with important implications for learning and memory formation and for diseases such as AD. Indeed, both memory and AD are influenced by non-genetic factors that accumulate over time (Miller and O’Callaghan, 2008). In this sense, epigenetics might store long-lasting information and provide a platform for accumulating hits over time. This idea has inspired the hypothesis of the Latent Early life Associated Regulation (LEARn) model (Lahiri et al., 2008). This model suggests that a series of harmful events throughout lifetime, from gestation to old age, could accumulate epigenetic marks that modify the expression probability of certain genes, which in turn might induce or accelerate the onset and development of AD. Whether this hypothesis will withstand further experimentations remains to be determined but, for the moment, it provides an attractive food for thoughts.

## Author Contribution

JVSM and JG conceptualized and wrote the review.

## Conflict of Interest Statement

The authors declare that the research was conducted in the absence of any commercial or financial relationships that could be construed as a potential conflict of interest.
